# An innovative approach to increase viral hepatitis diagnoses and linkage to care using opt-out testing and an integrated care pathway in a London Emergency Department

**DOI:** 10.1371/journal.pone.0198520

**Published:** 2018-07-25

**Authors:** Hannah Evans, Sooria Balasegaram, Sam Douthwaite, Laura Hunter, Ranjababu Kulasegaram, Terry Wong, Antonio Querol-Rubiera, Gaia Nebbia

**Affiliations:** 1 United Kingdom Field Epidemiology Training Programme, Public Health England, United Kingdom; 2 Field Epidemiology Service, National Infection Service, Public Health England, London, United Kingdom; 3 Department of Infection, Guy’s and St Thomas’ NHS Trust, London, United Kingdom; 4 Emergency Department, Guy’s and St Thomas’ NHS Trust, London, United Kingdom; 5 Department of HIV/GU Medicine, Guy’s and St Thomas’ NHS Trust, London, United Kingdom; 6 Gastroenterology and Hepatology Department, Guy’s and St Thomas’ NHS Trust, London, United Kingdom; Centre de Recherche en Cancerologie de Lyon, FRANCE

## Abstract

Therapies that halt progression of chronic hepatitis B virus (HBV) and achieve a cure for chronic hepatitis C virus (HCV) have encouraged development of innovative strategies to diagnose and link patients to care. We describe the prevalence and risk factors for HBV and HCV infections and use of an opt-out hepatitis testing and integrated linkage to care pathway in a London Emergency Department (ED). ED patients aged ≥16 years having routine blood tests from 15 February-28 March 2016 were tested for hepatitis, unless opted out. Hepatitis B surface antigen (HBsAg) and hepatitis C antibody tests (HCV-Ab, including a confirmatory hepatitis C antigen test (HCV-Ag)) were pre-selected on electronic blood test requests. Linkage to care (attending one clinic appointment) was offered to HBsAg and HCV-Ag patients (new or known-disengaged with care diagnoses). Weighted prevalence estimates and risk factors for seropositivity adjusted by demographics and survey weights were calculated using logistic regression. Hepatitis testing uptake was 56% (3,290/5,865). Overall, 26 HBsAg (10 new diagnoses) and 63 HCV-Ab patients were identified of which 32 were HCV-Ag positive (10 new diagnoses). Weighted seroprevalence of HBsAg was 0.50% (95% CI 0.3–0.8%); HCV-Ab 2.0% (95% CI 1.5–2.7%) and HCV-Ag 1.2% (95% CI 0.8–1.7%). Risk factors for infection were being male (HBsAg: aOR 4.1, 95% CI 1.5–11.3), of non-White British ethnicity (HBsAg: aOR>11) or being homeless (HCV-Ag: aOR 18.9, 95% CI 6.9–51.4). We achieved a high linkage to care uptake for HBsAg (93%) and HCV-Ag (78%) among patients who were contacted and required linkage. A pre-selected hepatitis testing ordering system facilitated a high testing uptake. New and disengaged with care diagnoses and a high HCV prevalence were identified demonstrating the potential to identify and link patients to care in this setting. Strategies connecting clinical care with community outreach services are key for improving patient linkage to care.

## Introduction

Hepatitis B (HBV) and C (HCV) virus infections continue to cause considerable morbidity and mortality worldwide and within the UK. In the UK, an estimated 180,000 and 214,000 people have chronic HBV and HCV infection, respectively [1, 2]. In England, the highest number of HBV and HCV infections occur within London and within specific populations e.g. people who inject drugs (PWID), black and minority ethnic populations, prisoners, men who have sex with men (MSM), homeless people and migrants from highly endemic countries [3, 4]. Prevalence estimates for HCV antibody (HCV-Ab) and HBV (hepatitis B surface antigen, HBsAg) have been reported for England (HCV-Ab: 0.67% for those aged 15–59 years), for specific ethnic groups (South Asian, HBsAg: 1.2%, HCV-Ab: 1.6%) and for antenatal women in London (HBsAg: 0.8%) using modelling and routine surveillance data [5–7]. However viral hepatitis prevalence estimates of local and general populations in England and London are lacking and are needed to target future diagnostic and treatment services.

Delayed diagnosis of HBV and HCV infections is common with an estimated 40% of HCV infections in London remaining undiagnosed [4, 8]. Treatment uptake among HCV infected populations is low, with an estimated 17% receiving treatment between 2006 and 2011 in England [9, 10]. Sequelae of untreated chronic infections include development of cirrhosis or hepatocellular carcinoma resulting in a significant health and economic burden [11]. Recent developments in HCV therapy offering clearance of infection and effective treatments to reduce disease progression of HBV have driven an increasing demand to test, diagnose and link patients into appropriate treatment and care services [12, 13]. This would contribute to identifying undiagnosed patients, improving clinical outcomes and reducing onward transmission.

The 2016 Global Viral Hepatitis Strategy prioritised the need for innovative testing strategies with efficient linkage to care pathways with the aim to eliminate viral hepatitis as a public health threat by 2030 [14]. Currently in the UK, the National Institute for Health and Care Excellence (NICE) recommends HBV and HCV testing for at risk groups with the main settings for testing including general practices and specialist healthcare settings such as drug services, prisons, sexual health clinics and antenatal clinics [15]. Emergency Departments (EDs) have been identified as a potential candidate to offer HIV and blood borne virus screening to patients having blood tests taken as part of routine care and have been described elsewhere as a feasible and acceptable service model among staff and patients [16–18]. EDs may also be accessed more frequently by populations at increased risk of HCV infection as shown in one US study [19]. High prevalence estimates of HIV, HBV and HCV in ED populations have been reported in several European studies including a short-term ‘Going Viral’ campaign in nine UK EDs [17, 20–22]. Therefore, EDs may provide a valuable setting to offer opportunistic viral hepatitis testing to identify undiagnosed infections.

We describe the prevalence and risk factors of HBV and HCV infection during a six week pilot study in one London ED offering opt-out hepatitis testing and use of an integrated linkage to care pathway. The provision of these hepatitis services are in addition to a well-established routine HIV testing and follow-up programme which will not be analysed in this manuscript. This pilot study provides important local prevalence estimates and valuable evidence to inform local policies on whether routine HBV and HCV screening in EDs with an integrated clinical care pathway should be considered for future practice.

## Methods

### Study design, setting and participants

We conducted a prospective prevalence study at Guy’s and St Thomas’ NHS Foundation Trust (GSTFT) ED between 15 February 2016 and 28 March 2016. All patients aged 16 years or older having blood tests (full blood count/liver function tests) as part of their routine care were verbally informed by the clinician that a viral hepatitis test would be carried out unless they declined testing (opted-out). Parental consent was not required for patients aged 16 or 17 years old as per Department of Health consent guidance [23]. Posters and information leaflets outlining the intention to test, the testing process and provision of care for positive HBV and HCV patients were available throughout the ED and provided to the patient by the clinician. The electronic patient record (EPR) system was modified so that blood test orders for full blood count and liver function tests were pre-selected with HBV and HCV tests.

This project was a service evaluation study (http://www.hra-decisiontools.org.uk/research/) and did not require further ethical review by a NHS Ethics Committee or management permission through NHS Research & Development.

### Laboratory methods

The virology laboratory conducted HBV and HCV testing as per routine local protocol due to the benefits of performing viral hepatitis testing on a single blood sample and without incurring unsustainable costs. HBV infection was diagnosed upon the detection of Hepatitis B surface antigen (HBsAg) (Architect, Abbott Laboratories, Illinois), followed by a neutralization assay to confirm a reactive result. A reactive HBsAg test indicated HBV infection (acute or chronic). Testing for HCV infection consisted of an initial antibody screening test (Architect, Abbott Laboratories, Illinois). For reactive or equivocal HCV antibody (HCV-Ab) results, a HCV antigen test (HCV-Ag, Architect, Abbott Laboratories, Illinois) was performed (reflex testing) to confirm active HCV infection. For negative or equivocal HCV-Ag results, a second HCV-Ab assay was performed to confirm the presence of HCV antibodies.

### Operational definitions

Positive HBsAg and HCV-Ag patients were contacted and notified of their result by study investigators using contact details provided at ED attendance either directly (telephone or face-to- face), via their general practitioner, or by other healthcare services e.g. outreach homeless services. Diagnosis status (new, known or unknown) and engagement with care status at the time of diagnosis (engaged or disengaged) of HBsAg and HCV-Ag positive patients were identified upon contact with the patient or reviewing the EPR. For patients with a reactive HCV-Ab but negative HCV-Ag test result, indicating resolved infection, a letter was sent to their GP informing them of their result and the need to notify the patient for further follow-up.

### Linkage to care pathway

Linkage to care consisted of clinic appointments with a viral hepatitis nurse specialist or doctor, assessment of HCV RNA, viral load and genotyping data. Where possible, a liver FibroScan (Echosens, Paris, France) and ultrasound of the abdomen was also performed at the initial appointment in accordance with the European Association for Study of Liver (EASL) guidance [24, 25]. Only positive HBsAg and HCV-Ag patients that were newly diagnosed or were known but disengaged with care at the time of diagnosis were eligible for linkage to care. Patients requiring linkage were identified following clinical review of eligible patients. Two linkage to care outcomes were assessed; linked to care (attended one clinic appointment) and retained in care (attended >1 clinic appointments). Patients lost to follow-up at each stage of the linkage to care pathway were described. HCV and HBV treatments offered were in accordance with the National Institute for Health and Care Excellence (NICE) and NHS England guidelines [26, 27].

### Data collection and management

A dataset comprising all ED attendees, all patients who received routine blood tests, and all patients tested for HBsAg and/or HCV-Ab during the study period was extracted from EPR including demographic information (age, sex, ethnicity, residence postcode and GP postcode). Additionally, a linkage to care dataset recorded contact, diagnosis, engagement with care and linkage to care outcomes for each positive HBsAg and HCV-Ag patient. A unique patient identifier was used to create a collated dataset and enable analysis of each stage of the testing and linkage to care pathways. All data was handled in accordance with information governance policies to ensure patient confidentiality was maintained.

Duplicate ED attendances and patients aged below 16 years were removed from the dataset. Equivocal test results for HBsAg or HCV-Ab with a negative HCV-Ag were recoded as negative. Patients with unsuitable samples or samples not received in the laboratory were recoded as not tested. Patients with a record of an HBsAg or HCV-Ab test but no record of a blood test were recoded as having received a blood test to improve data completeness. For patients attending the ED multiple times during the study period, the record with a reactive hepatitis test was retained otherwise the earliest record was retained and updated with additional information from subsequent records to improve completeness, if required.

Age was recoded into a categorical variable and ethnicity reclassified into six categories.

ED arrival was analysed based upon arrival time (day: 08:00–19:59, night: 20:00–07:59) and arrival day (weekday: Monday-Friday, weekend: Saturday/Sunday). Socio-economic status of each patient was estimated by assigning a national deprivation quintile (Index of Multiple Deprivation 2015) to the patient residence postcode (or general practitioner postcode if unavailable or invalid). The Index of Multiple Deprivation measures relative deprivation in 32,844 small areas in England based on seven indicators of deprivation and ranked into quintiles from 1 (most deprived area) to 5 (least deprived area) to enable comparisons across quintiles [28]. A residence variable (no fixed abode/fixed abode) was created to assess homelessness. Patients with valid residence postcodes (full or partial) were assigned as having a fixed abode whereas patients with a postcode of ‘ZZ99 3VZ’ or of an accommodation for the homeless, as listed by the Homeless Link directory, or were identified at linkage to care to be homeless were assigned as having no fixed abode [29, 30].

### Statistical analysis

Demographic and ED arrival characteristics (day/night and weekday/weekend) of patients at each step of the testing and linkage to care pathways were described using numbers and proportions. Testing uptake of at least one hepatitis test among the blood tested population and linkage to care uptake (defined as attended one clinic appointment) among those requiring linkage was calculated. Crude and weighted prevalence estimates were calculated for each hepatitis test to enable inference to the wider blood tested population using sampling weights based on the age group, sex and ethnic group distribution of the blood tested population and 95% confidence intervals.

Univariable associations between risk factors (demographic and ED arrival variables), requiring a blood test and for being seropositive were investigated using logistic regression. All variables with a p value of ≤0.2 in the univariate analysis, as determined by the likelihood ratio test (LRT), were included in a multivariable regression model. Age group, sex and ethnic group were considered *a priori* variables. A final model was identified using a backwards stepwise selection process, eliminating variables with the highest p values by LRT (including any *a priori* variables) and examining for possible confounders identified by >10% change in the odds ratios. Sampling weights were applied to the final model to obtain adjusted weighted odds ratios for positivity, 95% confidence intervals and Wald p values.

## Results

A total of 13,179 patients attended the ED after exclusion of 1,649 records due to; patients aged below 16 years (n = 31), duplicate entries (n = 68) and multiple ED attendances (n = 1,550). Information on sex was missing for one patient. Records were recoded due to; no blood test record for patients tested for hepatitis (n = 24), equivocal test results (n = 8) or insufficient or unsuitable samples (n = 31).

ED attendees were predominantly female, aged 30–49 years, of White British ethnicity, attended during weekdays or during the day and where information was available, resided in the two most deprived quintiles (Table 1). Older age and ED arrival at night were associated with an increased odds of having a blood test whereas being male, of White other ethnicity or arrival on the weekend were associated with a lower odds of having a blood test (Table 1).

**Table 1:** Demographics of ED attendees, blood tested patients and those tested for viral hepatitis, GSTFT, 15 February-28 March 2016.

Characteristic		ED attendees		Blood tested patients				Patients tested for HBsAg/HCV-Ab			
		n	%	n	%	aOR (95% CI)	P value	n	%	aOR (95% CI)	P value
Total		13,179	100	5,865	100			3,290	100		
Sex	Female	6,728	51	3,058	52	*Reference*		1,654	50	*Reference*	
	Male	6,450	49	2,807	48	0.9 (0.8–1.0)	0.002	1,636	50	1.2 (1.1–1.3)	0.002
Age (years)	16–29	4,124	31	1,294	22	*Reference*		720	22	*Reference*	
	30–49	4,882	37	1,973	34	1.5 (1.4–1.7)		1,145	35	1.1 (0.9–1.3)	
	50–69	2,691	20	1,449	25	2.5 (2.3–2.8)		822	25	1.0 (0.9–1.2)	
	70+	1,482	11	1,149	20	7.4 (6.4–8.5)	<0.001	603	18	0.8 (0.7–1.0)	0.009
Ethnicity	White British	4,768	36	2,377	41	*Reference*		1,376	42	*Reference*	
	White Other	2,981	23	1,083	18	0.7 (0.7–0.8)		597	18	0.9 (0.8–1.0)	
	Black/Black British	2,366	18	1,096	19	1.0 (0.9–1.1)		577	18	0.8 (0.7–0.9)	
	Asian	839	6	377	6	1.0 (0.8–1.1)		206	6	0.9 (0.7–1.1)	
	Mixed/Other	777	6	316	5	0.9 (0.7–1.0)		193	6	1.1 (0.9–1.4)	
	Unknown	1,448	11	616	11	0.9 (0.8–1.1)	<0.001	341	10	0.9 (0.7–1.0)	0.024
ED arrival day	Weekday	9,879	75	4,490	77	*Reference*		2,537	77		
	Weekend	3,300	25	1,375	23	0.9 (0.8–0.9)	<0.001	753	23		
ED arrival time	Day (08:00–19:59)	9,297	71	4,037	69	*Reference*		2,319	70	*Reference*	
	Night (20:00–07:59)	3,882	29	1,828	31	1.3 (1.2–1.4)	<0.001	971	30	0.8 (0.7–0.9)	0.001
IMD quintile area^a^	Q1 (most deprived)	4,217	34	1,957	35			1,092	35		
	Q2	4,660	37	2,084	37			1,139	36		
	Q3	2,137	17	938	17			548	17		
	Q4	855	7	342	6			201	6		
	Q5 (least deprived)	615	5	264	5			153	5		
Residence^b^	Fixed abode	12,656	98	5,647	98			3,165	98		
	No fixed abode	248	2	124	2			72	2		

aOR: adjusted odds ratio, HBsAg: hepatitis B surface antigen, HCV-Ab: hepatitis C antibody, Blood tested patients: Full blood count or liver function tested

^a^ Based on 12,484 patients following exclusion of 695 patients outside of the UK, invalid postcodes or had no fixed abode

^b^ Based on 12,904 patients following exclusion of 275 patients with unknown residence postcodes or area of residence or resident outside the UK.

### Uptake of hepatitis testing

Testing uptake was 56% (3,290/5,865) with 97% (3,187) of patients tested for both HBV and HCV infection. Of those tested; half were male, had a median age of 46 years (IQR 31–63 years), 42% (1,376) identified as White British and 71% (2,231) resided in the two most deprived quintiles (Table 1). Overall, 108 patients (3%) were tested for HBV or HCV more than once during the study period.

### Prevalence and risk factors for seropositivity

#### Hepatitis B

Overall, 26 HBsAg positive patients were identified from 3,265 patients tested for HBsAg, corresponding to an overall HBsAg prevalence of 0.8% (95% CI 0.5–1.2%). Prevalence was highest among those who were male (1.2%, 95% CI 0.8–1.8%), aged 30–49 years (1.2%, 95% CI 0.7–2.1%), of Black/Black British (1.9%, 95% CI 1.1–3.4%) or Mixed/Other ethnic background (2.1%, 95% CI 0.8–5.5%) or resided in the second most deprived quintile (1.2%, 95% CI 0.7–2.1) (Table 2). Weighted prevalence of HBsAg among the blood tested population was estimated as 0.5% (95% CI 0.3–0.8%). Factors significantly associated with being HBsAg positive were being male (adjusted OR (aOR) 4.1, 95% CI 1.5–11.3) or having an ethnicity other than White British (aOR>11). No differences in the odds of being positive for HBsAg were identified for age.

**Table 2:** Prevalence and risk factors for hepatitis B virus infection, GSTFT, 15 February–28 March 2016.

Characteristic		HBV (HBsAg)				
		Tested	Positive	Prevalence	Adjusted weighted odds of positivity	
		n	n	%(95% CI)	aOR(95% CI)	*P* value^c^
Crude estimates		3,265	26	0.8 (0.5–1.2)		
Weighted estimates		3,265	26	0.5 (0.3–0.8)		
Sex	Female	1,646	7	0.4 (0.2–0.9)	*Reference*	
	Male	1,619	19	1.2 (0.8–1.8)	4.1 (1.5–11.3)	0.007
Age (years)	16–29	715	2	0.3 (0.1–1.1)	*Reference*	
	30–49	1,135	14	1.2 (0.7–2.1)	3.0 (0.6–15.4)	
	50–69	813	5	0.6 (0.3–1.5)	1.8 (0.3–10.9)	
	70+	602	5	0.8 (0.4–2.0)	4.6 (0.7–28.5)	0.34
Ethnicity	White British	1,367	1	0.1 (0.0–0.5)	*Reference*	
	White Other	594	4	0.7 (0.3–1.8)	11.4 (1.5–88.4)	
	Black/Black British	572	11	1.9 (1.1–3.4)	36.9 (5.6–244.8)	
	Asian	204	2	1.0 (0.3–3.8)	15.1 (1.4–159.8)	
	Mixed/Other	191	4	2.1 (0.8–5.5)	48.1 (6.4–358.7)	
	Unknown	337	4	1.2 (0.5–3.1)	23.1 (3.1–172.7)	0.003
ED arrival day	Weekday	2,522	20	0.8 (0.5–1.2)		
	Weekend	743	6	0.8 (0.4–1.8)		
ED arrival time	Day (08:00–19:59)	2,299	17	0.7 (0.5–1.2)		
	Night (20:00–07:59)	966	9	0.9 (0.5–1.8)		
IMD quintile area^a^	Q1 (most deprived)	1,085	9	0.8 (0.4–1.6)		
	Q2	1,131	14	1.2 (0.7–2.1)		
	Q3	542	3	0.6 (0.2–1.7)		
	Q4	199	0	0		
	Q5 (least deprived)	152	0	0		
Residence^b^	Fixed abode	3,140	26	0.8 (0.6–1.2)		
	No fixed abode	72	0	0		

HBV, hepatitis B virus; HBsAg, hepatitis B surface antigen; aOR, adjusted weighted odds ratio; CI, confidence interval

^a^ Based on 3,109 patients following exclusion of 156 patients outside of the UK, invalid postcodes or were had no fixed abode

^b^ Based on 3,212 patients following exclusion of 53 patients with unknown residence postcodes or area of residence or resident outside the UK.

^c^ *p* values were calculated using the Wald test

#### Hepatitis C

Of 3,212 patients tested for HCV-Ab, 63 HCV-Ab and 32 HCV-Ag positive patients were identified (Table 3). Nine HCV-Ab positive patients were not tested for HCV-Ag due to prior knowledge of antigen status. Crude HCV-Ab prevalence was 2.0% (95% CI 1.5–2.5%) and HCV-Ag prevalence was 1.0% (95% CI: 0.7–1.4%). HCV-Ag prevalence was highest among those who were male (1.5%, 95% CI 1.0–2.3%), aged 30–69 years (>1.2%), of White British or White Other ethnicity (>1.2%), attended during the weekend (1.2%, 95% CI 0.6–2.4%) or at night (1.4%, 95% CI 0.8–2.4%) or had no fixed abode (13.4%, 95% CI 7.1–23.9%). Weighted prevalence estimates for HCV-Ab among the blood tested population was 2.0% (95% CI 1.5–2.7%) and for HCV-Ag was 1.2% (95% CI 0.8–1.7%). No fixed abode was strongly associated with being HCV-Ag positive (aOR 18.9, 95% CI 6.9–51.4).

**Table 3:** Prevalence and risk factors for hepatitis C virus infection, GSTFT, 15 February–28 March 2016.

Characteristic		HCV antibody (HCV-Ab)			HCV antigen (HCV-Ag)^a^			
		Tested	Positive	Prevalence	Positive	Prevalence	Adjusted weighted odds of positivity	
		n	n	% (95% CI)	n	% (95% CI)	aOR (95% CI)	*P* value^d^
Crude estimates		3,212	63	2.0 (1.5–2.5)	32	1.0 (0.7–1.4)		
Weighted estimates		3,212	63	2.0 (1.5–2.7)	32	1.2 (0.8–1.7)		
Sex	Female	1,633	17	1.0 (0.7–1.7)	8	0.5 (0.3–1.0)	*Reference*	
	Male	1,579	46	2.9 (2.2–3.9)	24	1.5 (1.0–2.3)	1.7 (0.7–4.3)	0.28
Age (years)	16–29	686	5	0.7 (0.3–1.7)	4	0.6 (0.2–1.5)	*Reference*	
	30–49	1,109	33	3.0 (2.1–4.2)	15	1.4 (0.8–2.2)	1.5 (0.5–5.0)	
	50–69	815	19	2.3 (1.5–3.6)	10	1.2 (0.7–2.3)	1.5 (0.4–5.7)	
	70+	602	6	1.0 (0.5–2.2)	3	0.5 (0.2–1.5)	0.6 (0.1–3.5)	0.60
Ethnicity	White British	1,331	26	2.0 (1.3–2.9)	17	1.3 (0.8–2.1)		
	White Other	583	17	2.9 (1.8–4.6)	7	1.2 (0.6–2.5)		
	Black/Black British	568	3	0.5 (0.2–1.6)	3	0.5 (0.2–1.6)		
	Asian	203	2	1.0 (0.3–3.9)	1	0.5 (0.1–3.4)		
	Mixed/Other	193	3	1.6 (0.5–4.7)	0	0		
	Unknown	334	12	3.6 (2.1–6.2)	4	1.2 (0.5–3.2)		
ED arrival day	Weekday	2,478	44	1.8 (1.3–2.4)	23	0.9 (0.6–1.4)		
	Weekend	734	19	2.6 (1.7–4.0)	9	1.2 (0.6–2.4)		
ED arrival time	Day (08:00–19:59)	2,278	38	1.7 (1.2–2.3)	19	0.8 (0.5–1.3)		
	Night (20:00–07:59)	934	25	2.7 (1.8–4.0)	13	1.4 (0.8–2.4)		
IMD quintile area^b^	Q1 (most deprived)	1,066	12	1.1 (0.6–2.0)	6	0.6 (0.3–1.3)		
	Q2	1,124	23	2.1 (1.4–3.1)	13	1.2 (0.7–2.0)		
	Q3	533	6	1.1 (0.5–2.5)	2	0.4 (0.1–1.5)		
	Q4	191	0	0	-	-		
	Q5 (least deprived)	143	0	0	-	-		
Residence^c^	Fixed abode	3,089	42	1.4 (1.0–1.9)	22	0.7 (0.5–1.1)	*Reference*	
	No fixed abode	71	20	28.1 (19.0–39.7)	9	13.4 (7.1–23.9)	18.9 (6.9–51.4)	<0.001

HCV, hepatitis C virus; aOR, adjusted weighted odds ratio; CI, confidence interval

^a^ Denominator based on 3,203 patients following exclusion of patients that were not tested for HCVAg due to prior knowledge of HCVAg status

^b^ Based on 3,057 hepatitis C antibody or 3,052 hepatitis C antigen patients following exclusion of patients outside of the UK, invalid postcodes or had no fixed abode

^c^ Based on 3,160 hepatitis C antibody or 3,151 hepatitis C antigen patients following exclusion of patients with unknown residence postcodes or area of residence or resident outside the UK.

^d^ *p* values were calculated using the Wald test

No patients were co-infected with HBV and HCV infection.

### Linkage to care outcomes

#### Hepatitis B

**Fig 1:**
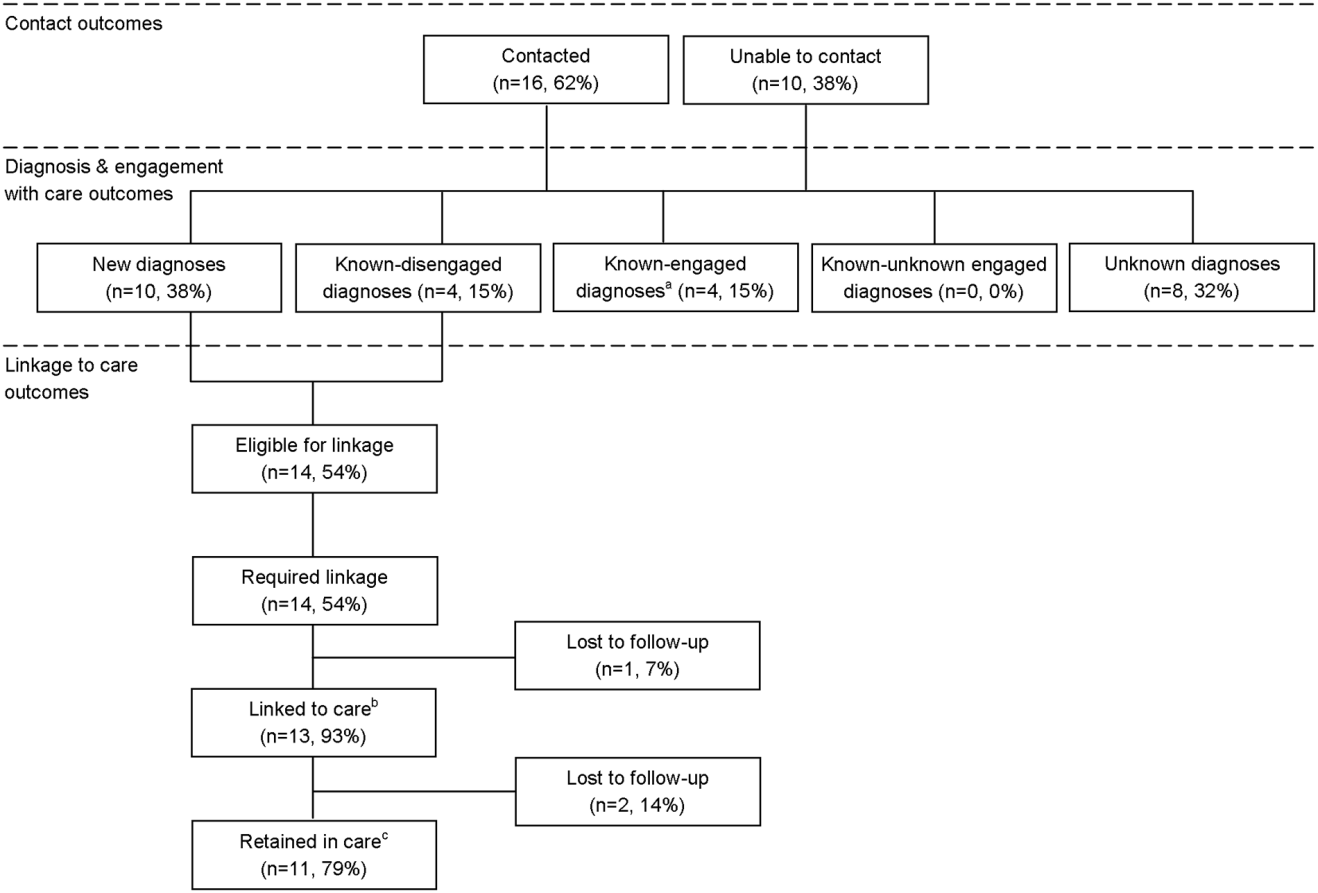
A flow chart of the linkage to care pathway and outcomes for positive hepatitis B surface antigen patients (n = 26). ^a^ Includes two not contacted but identified on EPR. ^b^ Attended one clinic appointment. ^c^ Attended ≥1 clinic appointments.

Of the 26 HBsAg positive patients, 16 (62%) were able to be contacted and notified of their test result (Fig 1). A total of 10 (38%) new diagnoses and eight (31%) known diagnoses (four currently engaged with care and four disengaged with care) of HBV infection were identified. Overall, 14 (54%) HBsAg patients required linkage to care and were provided a clinic appointment; 13 were linked to care (linkage to care uptake 93%) and 11 (79%) were retained in care (Fig 1). All HBsAg positive patients were diagnosed with chronic infection. One HBsAg patient died at 12 months of an unrelated cause. Of the 11 retained patients, eight (73%) received a FibroScan and ultrasound abdomen within their second appointment, three (27%) received an ultrasound abdomen only. Two patients were eligible for HBV treatment and were started on either Entecavir or Tenofovir treatment. All other patients had evidence of inactive hepatitis B infection.

#### Hepatitis C

**Fig 2:**
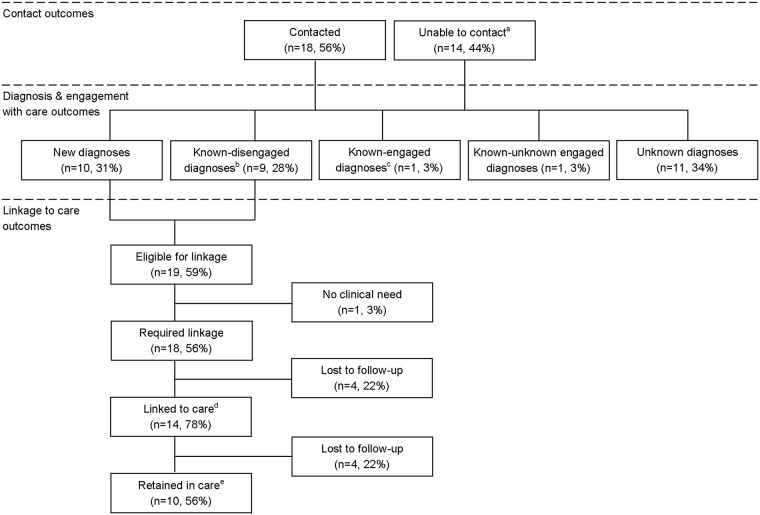
A flow chart of the linkage to care pathway and outcomes for hepatitis C antigen patients (n = 32). ^a^ Includes two patients actively not contacted. ^b^ Includes one re-infected patient and one not contacted but identified on EPR. ^c^ Not contacted but identified on EPR. ^d^ Attended one clinic appointment. ^e^ Attended ≥1 clinic appointments.

Of the 32 HCV-Ag patients, 18 (56%) were contacted and notified of their test result (Fig 2). A total of 10 (31%) new diagnoses and 11 (34%) known diagnoses of HCV infection including one re-infected patient were identified. Among the 11 known diagnoses, nine were disengaged with care. A total of 18 (56%) patients required linkage to care and were provided a clinic appointment; 14 were linked to care (linkage to care uptake 78%) and 10 (56%) were retained in care. Of the 10 patients retained in care, nine were HCV genotype 1 (five subtype 1a, four subtype 1b) and for one patient no RNA was detected. Among the 10 retained patients, six (60%) received a FibroScan of which four patients had a FibroScan score above seven, indicating significant fibrosis. One additional patient had a FibroScan with a score above 7. Five retained patients started treatment of which four completed treatment and cleared infection and one self-discontinued treatment without clearing infection.

## Discussion

This study is one of few inner-city studies to have reported the prevalence, risk factors and linkage to care outcomes of HBV and HCV infections in an ED setting using an opt-out testing and integrated clinical care pathway strategy. We found a crude prevalence of HBsAg, HCV-Ab and HCV-Ag of 0.8%, 2.0% and 1.0%, respectively. Risk factors for infection were being male (HBsAg), of non-White British ethnicity (HBsAg) or having no fixed abode (HCV-Ag). During the study, we detected 10 new diagnoses of each viral hepatitis infection and achieved a high linkage to care uptake for eligible HBsAg (93%) and HCV-Ag (78%) patients. We did not identify any individuals co-infected with HBV and HCV infection in this study. Potential reasons for this finding may be due to the small sample size given the short six week study period, positive patients (including those co-infected) declined testing or due to the different populations that these infections predominantly occur within.

Our crude HBsAg prevalence estimate was higher than reported in a Dublin ED (0.49%) but similar to that reported across nine UK EDs (0.71%) [17, 20]. The lack of general and local population prevalence estimates for HBV makes it difficult to draw comparisons with our weighted HBsAg prevalence estimate of 0.5% reflecting the prevalence in the blood tested population. However, a HBsAg prevalence of 0.8% among antenatal women (aged 16–49 years) in London compared to 0.5% of women in the same aged population in this study potentially indicates a lower HBsAg prevalence in this ED cohort [7]. We observed a low proportion (27%) of positive female HBsAg patients potentially reflecting more women already aware of their status due to the antenatal hepatitis B screening. It also indicates that the ED setting may provide a good opportunity to identify HBV infections in men who are not otherwise tested in any screening programme. Risk factors for HBV infection identified in this study e.g. being male, aged 30–49 years or in specific ethnic groups (Black/Black British and Mixed/Other) match those reported nationally which is expected given that the disease burden in England is disproportionately driven by infections in London [3, 31]. Although routine hepatitis B vaccination was recently introduced into the UK childhood vaccination schedule in August 2017, following this study, its impact on reducing hepatitis B-associated morbidity and mortality may not be apparent in the near future due to the majority of hepatitis B transmission occurring in adulthood [32].

Few studies have reported prevalence estimates of active hepatitis C infection (HCV-Ag) which is crucial for targeting clinical efforts to reduce onward transmission. Although there is a lack of UK literature to enable comparisons, our crude prevalence of HCV-Ag was slightly lower compared to a previous ED study in Germany (1.6%, 95% CI 1.5–1.8%) using RNA testing [21]. In relation to HCV-Ab, our crude prevalence estimate was comparable to estimates reported in EDs in the UK (1.84%) but lower than observed in Germany (2.6% 95% CI 2.4–2.8%), Switzerland (2.7%, 95% CI 2.3%-3.2%) and approximately half the prevalence reported in Dublin (5.05%)[17, 20–22]. In comparison to modelled estimates of HCV-Ab prevalence in the general population in England (aged 15–59 years: 0.67%, 95% CI 0.50–0.94%), our weighted prevalence of HCV-Ab was over three times higher (16–59 years: 2.5%) [5]. Possible reasons for the high HCV prevalence may reflect accessing high risk groups such as PWIDs and homeless people who may not routinely engage with other healthcare services offering testing. Based upon the 2015 Unlinked Anonymised Monitoring Survey of PWIDs, 55% of PWIDs in London were HCV antibody positive and an estimated 90% of HCV diagnoses in England are attributable to injecting drug use [33, 34]. Our results indicate a high prevalence of HCV in this ED cohort, suggesting the ED may be a good setting to offer hepatitis testing opportunistically to those with poorer access to healthcare.

This pilot study demonstrated the potential for HCV antigen testing as a reflex test to discriminate between active or past infection for seropositive individuals. HCV antigen testing has been suggested to provide a cheaper and more rapid turnaround time to issue results compared to RNA testing [35]. However, use of HCV core antigen testing as a reflex test (i.e. following a reactive HCV-Ab test) rather than as the initial screening assay, may have missed a small proportion of patients with chronic HCV with low viral load and therefore results should be interpreted with this testing strategy in mind [21]. Use of HCV antigen testing may be an important component for HCV screening enabling the potential for earlier diagnosis, linkage to care and commencement of treatment [35, 36].

A key strength of this study was the achievement of a high testing uptake (56%) in comparison to similar studies where uptake ranged from 27–50% [17, 20]. We propose that the use of an opt-out testing strategy and the efficiency in electronic pre-selected ordering of hepatitis tests likely facilitated the high testing uptake. In addition, delivery of free healthcare in the UK likely contributed to a high testing uptake and willingness to access care. Replication of this ED testing and linkage to care strategy in other European countries where healthcare is fee-based may influence the willingness of ED patients to accept testing and if continued care is fee-based. Although feedback from ED staff was not obtained in this study, staff experience in a Dublin study found that opt-out testing enabled easier viral hepatitis screening due to the elimination of risk assessment prior to testing [17].

The establishment of an integrated linkage to care pathway for this study enabled streamlined access of infected individuals into care. Although a high linkage to care uptake was obtained, approximately a third of patients were unable to be contacted and therefore potentially remain unaware of their diagnosis. There were also some difficulties in maintaining engagement with care. Often these not contactable and lost to follow up patients were within the PWIDs or homeless populations, as identified on patient records. Interventions to increase contact, linkage to care and treatment uptake among homeless and PWID populations are required to provide better clinical outcomes. Strategies currently being investigated in this acute trust include; adding an alert to the electronic patient record to inform clinicians of the diagnosis for patients re-attending the ED, establishing a close working network with outreach and community teams who engage with these populations and offering walk-in clinics for follow-up.

This study has some important limitations including differences identified in some populations being tested and not tested for viral hepatitis. Unfortunately, the reason for non-testing was not captured in this study and therefore we were unable to differentiate between those who were not tested because they were not offered a hepatitis test or had opted-out for example due to hepatitis status being known. Knowledge of reasons for non-testing would be informative for improving the offer of testing particularly if they are modifiable factors. Furthermore, as we calculated weighted prevalence estimates based on the assumption that the tested and not tested populations are similar, there may have been important unmeasured differences between the two groups. Consequently, we are unable to determine the influence of this selection bias on the direction of the prevalence estimates. Findings on risk factors for seropositivity should be interpreted with some caution as important risk factors such as injecting drug use history, country of birth, sexual behaviour and prison history were unable to be measured in this study. Although efforts were undertaken to account for homelessness using postcode data and could be considered to some extent accounting for injecting drug use as an estimated 39% of homeless people reported drug use or recovering from a drug problem in a 2014 Homeless Health Needs Audit in England, it is likely we underestimated the proportion of homeless people and the method may be prone to misclassification bias [37]. An indication on the direction of this bias on prevalence estimates is unknown.

## Conclusion

We conclude that an adaptable electronic testing ordering system and the adoption of an opt-out testing strategy are key components to assist viral hepatitis testing uptake in an ED. During the six week pilot study, 20 new viral hepatitis diagnoses were detected which may have been otherwise missed providing a benefit to the patient through earlier diagnosis and improved clinical outcome and to public health by reducing onward transmission. This study also provided the opportunity to identify and re-engage known diagnoses into care using a more streamlined linkage to care pathway. To improve the testing process, future ED viral hepatitis screening programmes should consider implementing an alert on the patient record system to identify patients recently tested for hepatitis and to explore the feasibility of recording information on PWID status, sexual behaviour and reason for patient refusal for testing to better understand the local prevalence of viral hepatitis in this population. We also recommend the need to establish a dedicated linkage to care coordinator with strong relationships with outreach services to homeless and PWID communities to contribute to a successful linkage to care pathway. This study provided valuable local prevalence estimates however a longer term study in the same London ED is currently being undertaken incorporating the improved testing and linkage to care strategies described above and a cost-effectiveness evaluation to better inform local hepatitis diagnosis and management policies in the future.
